# Supplementary material for: Dynalign II: common secondary structure prediction for RNA homologs with domain insertions
Source: Nucleic Acids Res. 2014 Nov 21;42(22):13939–48. doi: 10.1093/nar/gku1172 (PMC4267632; doi:10.1093/nar/gku1172)
Supplement: SUPPLEMENTARY DATA [file supp_gku1172_nar-02021-z-2014-File012.zip › manual/Overview/Makefiles.html]

RNAstructure Installation and Overview: Makefiles


|  |  |  |
| --- | --- | --- |
|  | RNAstructure Installation and Overview Makefiles | - Contents - Index |
| Quick Navigation  Go to: Main Makefile \| JAVA Interface Makefile \| NAPSS Makefile \| OligoWalk Makefile \| PARTS Makefile \| Postscript Library Makefile \| RNA Class Makefile \| Testing Makefile \| TurboFold Makefile   ---   Main Makefile (Location: RNAstructure/)  Summary  The main Makefile's purpose is to build the text (command line) interfaces from the RNAstructure repository. It has a variety of convenient targets to do so, as well as certain general targets that provide useful information or moving functionality related to the interface executables.  Section 1: Included Definitions  At the top of the main Makefile, two include statements incorporate all variables defined in RNAstructure/compiler.h and RNAstructure/library\_defines.h.  Section 2: General Build Actions  There are three general build actions in the main Makefile, defined as follows:  (make) instructions  The "make instructions" target is a convenience target. It prints specific information on standard output to explain which targets can be used to create interfaces in the main Makefile. This information does not include all possible targets used in the Makefile; only those that, when directly invoked, build a particular interface.  (make) all  The "make all" target calls all the individual targets referenced in the "instructions" target, except for itself. The result of calling this target is that all the Linux/Unix/OS X text interfaces are built and placed with their libraries in the RNAstructure/exe directory. Note, however, that this target does NOT build the Windows text interfaces, the Windows GUI, or the JAVA cross platform GUI.  (make) install  "make install" copies the contents of the RNAstructure/exe directory to /usr/local/RNAstructure, so "make all" must be run first. "make install" can only be run as root on Linux platforms because of file permisions. Note that it is not required to run "make install" to use RNAstructure; it is a convenience target.  Section 3: Common Objects For All Interfaces  At the current time, there is only one file that is used in all the text interfaces the main Makefile builds, which is a command line parser. Since there have been other common objects in the past, and may be more common objects in the future, this common object is in a section of its own.  Section 4: Common Objects For Certain Interfaces  This section containts objects and variables that are used in multiple text interfaces the Makefile builds, but not all of them. This section, like the one before it, is a convenience section to make individual interface targets cleaner later on.  Section 5: Library Dependencies and Variables  This section holds commands that, if run, will build the specific text interface libraries, as well as convenience variables that will group these libraries with common objects. These convenience variables are used extensively when building specific interfaces.  Sections 6 and Higher: Text Interface Building Targets  Each of these sections has a target in it to build a specific text interface. In general, each of these sections is set up with two aliased commands that can be used to build the appropriate interface, as well as one that builds the main interface dependency, if necessary. There are a few instances in which the interface requires more complex build steps, but this is explained inside a section where appropriate.  Final Section: Object and Executable Cleanup  The final section of the main Makefile is devoted to cleanup commands, one that removes all object files from the repository ("make clean") and one that removes object files and any executables in the RNAstructure/exe directory ("make realclean"). These cleanup targets do not just clean up executables and object files made by the commands in this Makefile. Instead, they clean up executables and object files wherever they are found in the RNAstructure repository.   ---  JAVA Interface Makefile (Location: RNAstructure/RNAstructure\_java\_interface/)  Summary  The JAVA Interface Makefile's purpose is to build the cross platform JAVA GUI interface. This Makefile creates two files when run to completion: a JAVA JAR archive file called RNAstructure.jar and a dynamic library of C++ code (the name of this library varies depending on the operating system, see the JAVA GUI library listing for details).  Section 1: Included Definitions  At the top of the main Makefile, two include statements incorporate all variables defined in RNAstructure/compiler.h and RNAstructure/library\_defines.h.  Section 2: Set Special GUI Flags  For the GUI dynamic library to compile properly, the INCLUDEPATH variables must be added to the compiling and linking flags. The number of include paths used, as well as the exact flags used, vary depending on the current operating system.  Section 3: Define Repeated Commands  This section holds repeated building commands that are used many times in the main build targets.   Note that this section will probably be eliminated in some future version of RNAstructure, once the interface is improved.  Section 4: Define Actions  This section holds the main building and cleaning actions for the JAVA GUI. The GUI can be built using either "make" or "make all."   Note that this section will probably split into further pieces and reorganized in some future version of RNAstructure, once the interface is improved.   ---  NAPSS Makefile (Location: RNAstructure/napss/)  Summary  The NAPSS Makefile's purpose is to build the NAPSS text interface. The main Makefile, when it builds the NAPSS interface, calls this Makefile explicitly.  Using the Makefile  NAPSS can be built by typing "make" or "make NAPSS."  Makefile Organization  The NAPSS Makefile is not divided into sections like most other Makefiles. At its top, it includes RNAstructure/compiler.h, which is then followed by two convenience macros for dependency organization. After these macros is the main NAPSS target, followed by all dependencies. At the end of the file is a clean target, which deletes any object files in the RNAstructure/napss or RNAstructure/src directories.  Important Usage Notice  This Makefile will probably be removed in the near future (merged into the main Makefile), coinciding with improvements to NAPSS.   ---  OligoWalk Makefile (Location: RNAstructure/oligowalk/src/)  Summary  The OligoWalk Makefile's purpose is to build the OligoWalk text interface. The main Makefile, when it builds the OligoWalk interface, calls this Makefile explicitly.  Section 1: Included Definitions and Convenience Macro  At the top of the OligoWalk Makefile, an include statement incorporates all variables defined in RNAstructure/compiler.h, and directly below that is a convenience macro that defines a dependency list.  Section 2: OligoWalk Targets  This section contains a "make all" target that gives information on the proper target to use for building OligoWalk, and two aliased targets that build OligoWalk when run.  Section 3: Dependency Lists  This section contains dependency lists for all the object files that make up the OligoWalk interface.  Final Section: Object and Executable Cleanup  The final section of the OligoWalk Makefile is devoted to cleanup commands, one that removes all object files from the oligowalk/src directory ("make clean") and one that removes those object files and the OligoWalk executable, if necessary, in the RNAstructure/exe directory ("make realclean").  Important Usage Notice  This Makefile will probably be removed in the near future (merged into the main Makefile).   ---  PARTS Makefile (Location: RNAstructure/PARTS/)  Summary  The PARTS Makefile's purpose is to build the PARTS text interface and its associated library. The main Makefile, when it builds the PARTS interface, calls this Makefile explicitly.  Section 1: Included Definitions  At the top of the PARTS Makefile, two include statements incorporate all variables defined in RNAstructure/compiler.h and RNAstructure/library\_defines.h.  Section 2: Convenience Variables  This section holds a small group of convenience variables that define necessary directories and the location of the main object.  Section 3: Rules for Object Groups  This section holds another group of convenience macros that defines all the PARTS dependencies as easy to use groups.  Section 4: Targets for the PARTS Library and Executable  This section contains three targets: one to build the PARTS Library, one to build the PARTS executable, and a "make all" target that is an alias for the PARTS executable target.  Final Section: Object and Executable Cleanup  The final section of the PARTS Makefile is devoted to cleanup commands, one that removes all object files from the PARTS directory and any of its subdirectories ("make clean") and one that removes those object files and the PARTS library or executable, if necessary, in the RNAstructure/exe directory ("make realclean").   ---  Postscript Library Makefile (Location: RNAstructure/src/PostscriptWriting/)  Summary  The Postscript Makefile's purpose is to build the Postscript Library.  Section 1: Included Definitions  At the top of the Postscript Makefile, two include statements incorporate all variables defined in RNAstructure/compiler.h and RNAstructure/library\_defines.h.  Section 2: Rules for Object Groups  This section holds a pair of convenience macros that define all the Postscript dependencies as easy to use groups.  Section 3: Define Build Actions  This section contains two targets, "make all" and "make Postscript\_Library," that can be used to build the Postscript Library. Note that it is also possible to build the library simply by typing "make."  Sections 4 and 5: Rules for Postscript Dependencies  These sections hold the the dependencies that each object file for the Postscript library needs. The only reason the last dependency is in a section of its own is because it depends on everything, including itself, so it's different from the rest.   ---  RNA Class Makefile (Location: RNAstructure/RNA\_class/)  Summary  The RNA Class Makefile's purpose is to build the RNA, HybridRNA, Dynalign, Dynalign-SMP, Oligo, Multilign, and Multilign-SMP libraries. It can also build working examples for basic functions in the RNA, HybridRNA, Dynalign, Dynalign-SMP, and Oligo libraries.  Section 1: Included Definitions  At the top of the RNA Class Makefile, two include statements incorporate all variables defined in RNAstructure/compiler.h and RNAstructure/library\_defines.h.  Section 2: JAVA Interface Re-Definitions  This section contains variables whose values change if the objects being built by the Makefile are destined for the JAVA interface. If objects are being prepared for the JAVA interface, extra compiler flags and some different progress monitoring objects are necessary.  Section 3: Internal Macros for Object Groups  This section holds the object groups necessary to build each of the libraries. Not every library has a specific object group, only those where repeating the object list multiple times would be cumbersome.  Section 4: Define Build Actions  This section contains targets to build all the libraries and examples described in the summary, as well as a "make all" convenience target that lists instructions telling which targets can be invoked for each library and example executable.  Sections 5 and Higher: Dependency Lists  Each of these sections, roughly divided by the libraries or executables they're included in, holds dependency lists for objects that make up libraries and examples.  Final Section: Object and Executable Cleanup  The final section of the RNA Class Makefile is devoted to cleanup commands, one that removes all object files from the RNA\_class directory and the RNAstructure/src directory ("make clean") and one that removes those object files and any libraries or executables built from this Makefile, if necessary, in the RNAstructure/exe directory ("make realclean").   ---  Testing Framework Makefile (Location: RNAstructure/tests)  Summary  The testing Makefile's purpose is to build (if necessary) and run the text interfaces, comparing the output of the interfaces to a group of accepted results to ensure that the interfaces are running properly. Note that this Makefile does not do the testing itself; it calls a main script called "NixScript" in the RNAstructure/tests/testFiles directory that in turn calls a group of small scripts when appropriate. In this sense, the Makefile is somewhat of a convenience wrapper.  Convenience Targets  The "make all" target calls all the individual targets that test each interface, sequentially in alphabetical order. All tests can also be run by simply typing "make," or by using three other possible aliases: "make runTests," "make tests," or "make testing."  Interface Testing Targets  Each text interface has two distinct testing targets that are aliases for each other. For every interface, one can use either the command "make [Interface executable name]" or "make tests/[Interface executable name]" to run specific tests.  General Testing Strategy   1. Run "make *Text\_Interface\_Name*" to run a particular interface's tests. 2. Each available option in the interface is tested separately. Progress is shown by progress messages that say "*TestType*started..." and "*TestType*finished." 3. If a test passes, it will print "Test Passed." If it encounters a possible failure, it will print "Possible Failure: *TestType*." 4. If a test encounters a possible failure, all files that were generated over the course of the test are placed in the tests/RNAstructure\_error\_tests directory. 5. The file named *TestType*\_*InterfaceOption*\_errors.txt holds any error output from the interface when running the test. 6. The file named *TestType*\_*InterfaceOption*\_diff\_output.txt holds information detailing any differences between the output of a particular test for a particular option, and its expected output. 7. Any files with "test\_output" in the name are results files generated from running the test. 8. In general, the presence of error files or diff output files shows that an unexpected change has been made to a program, which may signal a problem.  ---  TurboFold Makefile (Location: RNAstructure/TurboFold/)  Summary  The TurboFold Makefile's purpose is to build the TurboFold text interface and its associated library, as well as the TurboFold-smp text interface. The main Makefile, when it builds the TurboFold or TurboFold-smp interface, calls this Makefile explicitly.  Section 1: Included Definitions  At the top of the TurboFold Makefile, two include statements incorporate all variables defined in RNAstructure/compiler.h and RNAstructure/library\_defines.h.  Section 2: Convenience Target  This section holds a "make all" target that builds TurboFold and TurboFold-smp both at once.  Section 3: Rules for Object Groups  This section holds a group of convenience macros that define all the dependencies used in both TurboFold and TurboFold-smp.  Section 4: Targets for the TurboFold Library and Executable  This section contains targets for objects specific to the serial TurboFold executable and TurboFold library.  Section 5: Targets for the TurboFold-smp Library and Executable  This section contains targets for objects specific to the parallel TurboFold-smp executable and TurboFold-smp library.  Final Section: Object and Executable Cleanup  The final section of the TurboFold Makefile is devoted to cleanup commands, one that removes object files and one that removes those object files and the TurboFold libraries or executables, if necessary, in the RNAstructure/exe directory ("make realclean").   --- | | |
| Visit The Mathews Lab RNAstructure Page for updates and latest information. | | |
